# The differential response of cold-experienced *Arabidopsis thaliana* to larval herbivory benefits an insect generalist, but not a specialist

**DOI:** 10.1186/s12870-019-1943-3

**Published:** 2019-08-02

**Authors:** Jana Oberländer, Vivien Lortzing, Monika Hilker, Reinhard Kunze

**Affiliations:** 1 Freie Universität Berlin, Institute of Biology - Applied Genetics, Dahlem Centre of Plant Sciences, Albrecht-Thaer-Weg 6, 14195 Berlin, Germany; 2 Present address: University of Bern, Molecular Plant Physiology, Altenbergrain 21, CH-3013 Bern, Switzerland; 3 Freie Universität Berlin, Institute of Biology - Applied Zoology / Animal Ecology, Dahlem Centre of Plant Sciences, Haderslebener Str. 9, 12163 Berlin, Germany

**Keywords:** Plant stress response, Herbivore, Defense, Larval feeding, *Pieris brassicae*, *Mamestra brassicae*

## Abstract

**Background:**

In native environments plants frequently experience simultaneous or sequential unfavourable abiotic and biotic stresses. The plant’s response to combined stresses is usually not the sum of the individual responses. Here we investigated the impact of cold on plant defense against subsequent herbivory by a generalist and specialist insect.

**Results:**

We determined transcriptional responses of *Arabidopsis thaliana* to low temperature stress (4 °C) and subsequent larval feeding damage by the lepidopteran herbivores *Mamestra brassicae* (generalist), *Pieris brassicae* (specialist) or artificial wounding. Furthermore, we compared the performance of larvae feeding upon cold-experienced or untreated plants. Prior experience of cold strongly affected the plant’s transcriptional anti-herbivore and wounding response. Feeding by *P. brassicae*, *M. brassicae* and artificial wounding induced transcriptional changes of 1975, 1695, and 2239 genes, respectively. Of these, 125, 360, and 681 genes were differentially regulated when cold preceded the tissue damage. Overall, prior experience of cold mostly reduced the transcriptional response of genes to damage. The percentage of damage-responsive genes, which showed attenuated transcriptional regulation when cold preceded the tissue damage, was highest in *M. brassicae* damaged plants (98%), intermediate in artificially damaged plants (89%), and lowest in *P. brassicae* damaged plants (69%). Consistently, the generalist *M. brassicae* performed better on cold-treated than on untreated plants, whereas the performance of the specialist *P. brassicae* did not differ.

**Conclusions:**

The transcriptional defense response of *Arabidopsis* leaves to feeding by herbivorous insects and artificial wounding is attenuated by a prior exposure of the plant to cold. This attenuation correlates with improved performance of the generalist herbivore *M. brassicae*, but not the specialist *P. brassicae*, a herbivore of the same feeding guild.

**Electronic supplementary material:**

The online version of this article (10.1186/s12870-019-1943-3) contains supplementary material, which is available to authorized users.

## Background

Plants have evolved a plethora of mechanisms to cope with abiotic or biotic environmental stress (e.g. [1–4]). Attack by herbivorous insects is a major threat for plants as it can lead to rapid loss of leaf material and thus reduced photosynthetic capacity, often causing severe yield and fitness loss [5–7].

Plant defense responses induced by herbivore attack represent a strategy, which is mobilized only on demand [8, 9]. Inducible defense responses are associated with transcriptional regulation of many genes and shifts in phytohormone levels. Intensively studied key regulators of wounding and herbivore defense responses are the phytohormones jasmonic acid (JA), abscisic acid (ABA), salicylic acid (SA) and ethylene (ET), which are the backbone of the plant immune signaling network [10–15]. Fine-tuning of defense responses to different herbivores is achieved by crosstalk of these signaling pathways and may involve additional plant hormonal regulators like auxins, gibberellins, and brassinosteroids [13, 16].

In natural environments, plants are frequently exposed to simultaneously or consecutively occurring environmental stresses. Combined stresses typically provoke distinct transcriptome reprogramming and plant reactions, which are not simply due to additive effects of the single stresses [17–23]. In case of consecutively occurring environmental stress, plants can “memorize” a past stressful event and benefit from this memory by preparing themselves for a more effective response to upcoming stress. This process has been termed “priming” of a stress response by a past stress experience (reviewed in [24–27]).

Studies on priming of plant responses to insect herbivory especially focused on herbivore-related priming factors, which reliably indicate future herbivory [28]. For example, plant volatiles induced by herbivory and perceived by as yet undamaged plant tissue have been shown to serve as a reliable factor in preparing a plant for improved anti-herbivore defense [29–32]. Furthermore, insect egg depositions on leaves that indicate upcoming larval herbivory have been shown to prepare a plant for more effective defense against the hatching larvae [30]. Previous exposure of plants to herbivory-induced volatiles or to insect egg depositions are known to alter the transcriptional response to herbivory [33–39].

So far, only a few recent studies addressed the influence of an herbivory-unrelated, abiotic stress – especially drought – on the plant’s response to subsequent herbivory by including transcriptional and/or metabolic analysis (e.g. [40–42]). However, stressful conditions such as cold often precede plant attack by herbivorous insects, which usually need warm temperatures for their activities. A study by Firtzlaff et al. [43] examined how exposure of *Arabidopsis thaliana* to mild cold affects plant defense against later herbivory by the specialist *Pieris brassicae.* The study showed that a significant subset of cold-regulated genes maintained altered transcript levels even after 1 day of deacclimation. Larval feeding, which started 1 day after deacclimation, induced a different transcriptome in the previously cold-exposed than in previously untreated plants and showed a weakened response of defense genes. However, larval performance of the specialist *P. brassicae* was similar on cold-experienced and untreated plants [43]. These findings are in accordance with some other studies, which also revealed that host plants with attenuated plant defense capacity did not affect the extent of feeding damage inflicted by a specialized herbivorous insect [44] nor the herbivore’s performance [45].

Generalist and specialist herbivorous insects are known to exhibit different tolerances to plant defenses [46]. However, it is unknown as yet whether they are differentially affected by changes in plant defense that are due to prior exposure of plants to abiotic stress. Here we addressed the questions of whether a generalist insect herbivore shows different sensitivity to cold-mediated changes of feeding-induced host plant defense than a specialist*,* and if so, which transcriptional differences between cold-treated plants fed on by a generalist or a specialist insect may explain these ecological effects*.* As in our previous study [43], we used the butterflies *P. brassicae* and *Mamestra brassicae* and the Brassicacea *A. thaliana* as host plant. *Pieris brassicae* is specialized on glucosinolate-containing host plants [47], mostly from the Brassicaceae family. Like other *Pieridae* species it possesses highly specific enzymes for detoxification of the glucosinolates [48–50], which are typical secondary metabolites of the *Brassicales*. As generalist, we studied *Mamestra brassicae,* a moth whose larvae are polyphagous on over 70 plant species in 22 plant families, but exhibit a preference for *Brassica* crops [51]. In contrast to *P. brassicae*, *M. brassicae* detoxifies glucosinolates by general oxidizing enzymes (reviewed by [52]). Both lepidopteran species are active in Europe from early spring to late autumn [53, 54]. They may produce two to three generations per season until they hibernate in the soil as pupae. In the natural habitats of *M. brassicae* and *P. brassicae*, which largely overlap with that of *A. thaliana* (GBIF Secretariat: GBIF Backbone Taxonomy. Accessed via www.gbif.org/species/1920506 and www.gbif.org/species/3052436 on 01 June 2019), in spring and in autumn a succession of cold days followed by a warm period is common.

In a first approach, we compared performance of *M. brassicae* and *P. brassicae* on *A. thaliana* plants previously exposed to mild cold. We found that *M. brassicae* showed improved performance on cold-experienced plants, whereas *P. brassicae* larval performance was the same on cold-experienced and control plants, thus confirming our previous results with this latter species [43]. To elucidate the transcriptional basis of these different ecological effects, we compared the transcriptomes of cold-experienced plants exposed to feeding by the specialist, the generalist or to artificial wounding. Including the artificial wounding treatment allowed disentangling insect species-specific effects from wounding effects on the cold stress-reprogrammed plant transcriptome. We found that transcriptional responses of previously cold-exposed plants to specialist feeding, generalist feeding and artificial wounding differed.

Prior cold experience led to differential regulation of 360 *M. brassicae* feeding damage-responsive genes. In 98% of these the transcriptional response to feeding damage was attenuated. In contrast, the respective fraction of genes was smaller in artificially wounded (681 genes, 84% with attenuated response) and in *P. brassicae* feeding-damaged plants (125 genes, 69% with attenuated response). These transcriptional changes in conjunction with the larval performance data indicate that the generalist benefits from the cold-mediated attenuation of feeding-induced gene de-regulation, whereas the specialist does not.

## Results

### Generalist and specialist herbivores show different performances on cold-stressed and control plants

**Fig. 1:**
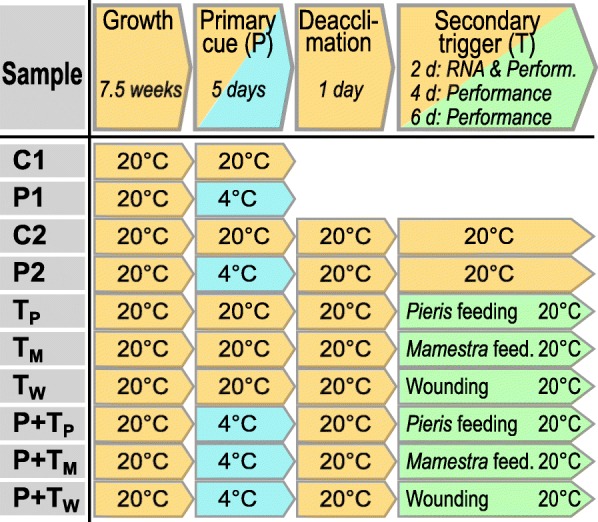
Experimental setup. Seven-week-old *Arabidopsis thaliana* Col-0 plants were subjected to either cold stress as primary (P) stimulus (4 °C, 5 days) or control (C) conditions (20 °C, 6 days). Plants treated with the primary stimulus were then retransferred to control conditions for 1 day (deacclimation phase). Subsequently plants were treated with a further triggering stimulus (T), i.e. with either larval feeding or artificial wounding. Plants which received both the P and T stimulus are here referred to as P + T plants. Plants, which were not exposed to cold and received only the T stimulus, are labelled as T plants. With respect to the T stimulus, we differentiate between T_P_ (feeding damage by *Pieris brassicae*)*,* T_M_ (feeding damage by *Mamestra brassicae*), and T_W_ (artificial wounding). Untreated control plants (C1, C2) remained at control conditions at 20 °C throughout the entire experiment

We exposed *A. thaliana* plants to cold (4 °C) for 5 days. After a deacclimation phase (20 °C) of 1 day, larvae of the generalist *M. brassicae* and the specialist *P. brassicae* were allowed feeding upon the previously cold-experienced plants or on control plants. The weight of these larvae on previously cold-treated (Fig. 1: P + T_P_ and P + T_M_) and untreated (Fig. 1: T_P_ and T_M_) plants and the extent of leaf damage inflicted by the larvae were compared.

**Fig. 2:**
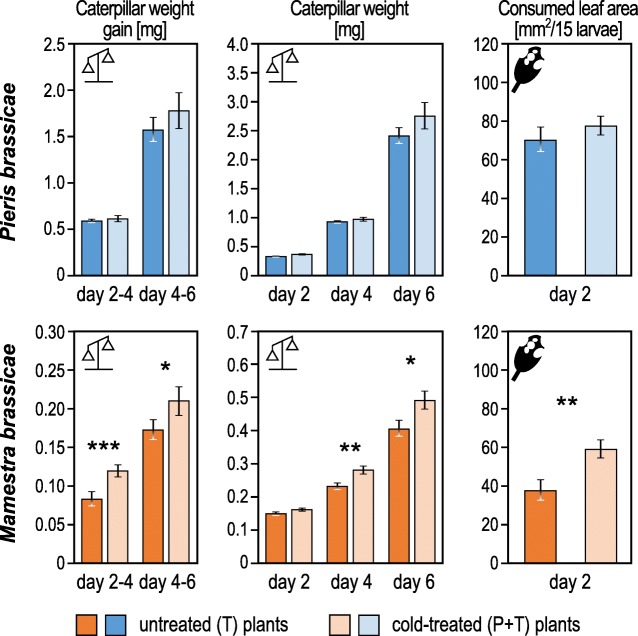
Performance of *Pieris brassicae* and *Mamestra brassicae* neonate larvae after 2, 4 and 6 days feeding upon previously cold-treated or untreated plants. Larvae were placed onto plants as neonates. Measured parameters (mean values ± SE) are caterpillar weight after 2, 4 and 6 days feeding, weight gain between day 2–4 and day 4–6, and consumed leaf area per plant (each with a group of 15 larvae) after 2 days feeding (from day 0 to day 2). Asterisks indicate significantly different values; *P < 0.05, **P < 0.01, ***P < 0.001 as calculated by Student’s *t*-tests. *N* (*P. brassicae*): T = 14 plants, P + T = 14 plants; *N* (*M. brassicae*): T = 11 plants, P + T = 11 plants

Weight gain and total weight of *P. brassicae* larvae, their leaf area consumption (Fig. 2) and the relative growth rate (RGR) of the larvae (Additional file 1: Figure S1) did not differ on previously cold-treated compared to untreated plants. In contrast, on previously cold-treated plants *M. brassicae* larvae consumed more leaf tissue, gained more weight and were heavier on these plants after a four- and six-day-feeding period than on untreated plants (Fig. 2). Accordingly, the RGR of the larvae was higher on cold-treated plants (Additional file 1: Figure S1). This observation suggests that the cold treatment alters either the metabolic status of the plants or their physiological reaction to leaf tissue damage in a way that is beneficial for the larval development of the generalist herbivore *M. brassicae*, but without consequences for the development of the specialist *P. brassicae*.

### Transcriptional response of *Arabidopsis* to feeding damage and artificial wounding

**Fig. 3:**
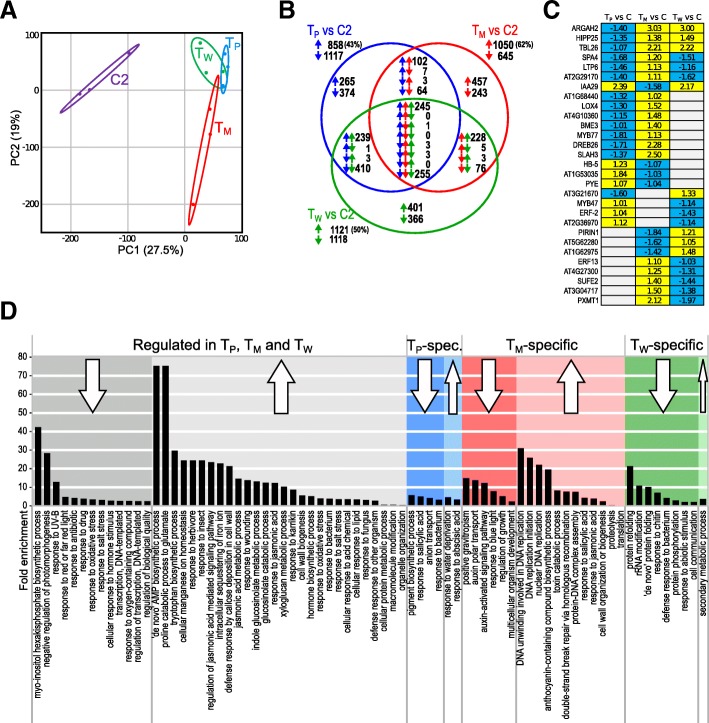
Regulation of gene expression in response to herbivory or artificial wounding in *Arabidopsis thaliana* leaves compared to untreated control leaves. Plants were exposed to feeding by *P. brassicae* larvae (T_p_), *M. brassicae* larvae (T_M_), artificial wounding (T_W_) or were left untreated (C2). Here, the T_P_, T_M_ and T_W_ treatments were adjusted in such a way that we obtained comparable extent of leaf damage (about 60 mm^2^ per plant; see Additional file 1: Figure S4). Plant material for microarray analysis was collected 2 days later. *N* = 3 biological replicates of each sample type. **a** Principle component analysis (PCA) of transcriptomic patterns of individual samples collected for microarray analysis. Samples originated from untreated control plants (C2, purple), feeding-damaged plants by either *P. brassicae* (T_P_, red) or *M. brassicae* (T_M_, blue) or artificially wounded plants (T_W_, green). The first two principal components, which explain most of the changes, are depicted (explained variances are shown at the axes). Ellipses indicate the 95% confidence interval. **b** The Venn diagram shows the number of genes, which were upregulated (upwards pointing arrows) or downregulated (downwards pointing arrows) in T_p_, T_M_ and T_W_ samples compared to C2 samples. **c** Heatmap depicting genes, which show opposed regulation in at least two treatments. Yellow = upregulated, blue = downregulated, grey = not regulated (log_2_ fold changes). **d** Gene Ontology terms associated with commonly or uniquely up- and downregulated genes

To investigate whether *Arabidopsis* plants respond differently to leaf damage by *P. brassicae* and *M. brassicae* larvae and to artificial wounding, we analyzed the transcriptomes in leaves from plants grown at 20 °C (Fig. 1, samples T_P_, T_M_, T_W_ and C2). A Principal Component Analysis (PCA) based on gene expression values of the differently treated plants revealed that the patterns of plants exposed to *P. brassicae* feeding, *M. brassicae* feeding and artificial wounding were clearly separated from untreated control samples. However, the patterns of the treated samples partially overlapped with each other, indicating that expression of a fraction of genes is similarly regulated in the treated samples (Fig. 3a).

Overall, 1975, 1695, and 2239 differentially expressed genes (DEGs) were identified that showed ≥2fold expression change after 2 days feeding damage by *P. brassicae* (T_P_ vs C2), *M. brassicae* (T_M_ vs C2) or wounding (T_W_ vs C2), respectively (Fig. 3b, Additional file 2: Table S1). As the majority of these genes responded qualitatively and quantitatively similarly to the three damage types (Additional file 1: Figure S2), the magnitude of the plant’s transcriptional response to herbivory or artificial wounding was similar. However, feeding damage by the generalist *M. brassicae* resulted in a larger fraction of upregulated genes (62% of 1695 genes in T_M_ vs C2; Fig. 3b) than by the specialist *P. brassicae* (43% of 1975 genes in T_P_ vs C2, Fig. 3b)*.* In total, 507 DEGs were regulated in all three sample types (central intersection in Fig. 3b). 176 DEGs specifically responded to larval feeding by either species but not to artificial wounding (Fig. 3b; intersection of T_P_ vs C2 and T_M_ vs C2 but not T_W_ vs C2), and 639, 700 and 767 genes were uniquely regulated upon *P. brassicae* feeding, *M. brassicae* feeding and artificial wounding, respectively. In the intersections, almost all DEGs (94–99%) were regulated in the same direction (Fig. 3b).

To disentangle common and unique regulated processes, an enrichment analysis of biological process-Gene Ontology (GO) terms was conducted (Fig. 3d). Among the 255 genes downregulated by all three damage types (Fig. 3b; central intersection), 13 GO terms were significantly enriched, which associate predominantly with responses to light, transcription, and growth. Among the 245 commonly upregulated genes, 28 GO terms were enriched, including several defense-related processes, such as response to and regulation of jasmonic acid, glucosinolate metabolism and response to insects, herbivores, bacteria, and fungi. These defense-related processes include many well described wounding- and feeding-responsive genes, i.e. *JAZ10, VSP1, VSP2, LOX2*, *CYP79B2*, *CYP79B3*, *IGMT1*.

Among the DEGs that were specifically responding to *P. brassicae-*feeding, two GO terms associated with abiotic stress were enriched in the 265 upregulated genes (“response to ABA”, “response to water deprivation”) and four GO terms were enriched in the downregulated genes, including the biological process “response to salicylic acid”.

Many *M. brassicae* feeding*-*specific upregulated genes fall into GO terms related to transcription and defense, including processes like “DNA replication initiation”, “response to jasmonic acid” and “response to salicylic acid”, whereas the six GO terms overrepresented among *M. brassicae*-specific downregulated genes are associated with development and growth.

Artificial wounding-specific responses were overall more generic. Out of 401 upregulated genes only one GO term (“secondary metabolic process”) consisting of 19 genes was weakly enriched. The eight GO terms associated with downregulated genes ranged from protein folding to “defense response to bacterium” to “response to abiotic stress”.

Feeding by *P. brassicae* evoked only a weak upregulation of two indole-glucosinolate biosynthesis genes, *CYP79B2* and *CYP79B3*, the indole-glucosinolate O-methyltransferases *IGMT1* and *IGMT5* [55, 56] and the nitrile specifier gene *NSP3* (Additional file 2: Table S1). Feeding by *M. brassicae* induced a stronger and more complex transcriptional response in the glucosinolate pathway. In addition to the *P. brassicae*-induced genes, *MYB51, NSP1*, *CYP81F2*, *CYP81F4* and *IGMT2* were upregulated. Yet, the strongest effects on the glucosinolate system, upregulation of indole-glucosinolate synthesis genes and nitrile specifier protein genes and downregulation of aliphatic glucosinolate synthesis genes, was observed upon artificial wounding of the leaf.

In total, 1648 genes were responsive to at least two damage types. The vast majority of these genes were regulated in the same direction, only 29 genes were regulated in opposite directions (intersections in Fig. 3b). The genes with highest regulation differences (15- to 21-fold difference) between at least two treatments were *ARGAH2*, *IAA29*, *SLAH3*, *DREB26,* and *PXMT1* (Fig. 3c). ARGAH2, one of two arginase proteins known in *Arabidopsis*, is involved in defense responses, as its expression is inducible by methyl jasmonate treatment [57]; this gene was clearly downregulated only by *P. brassicae* feeding, but not by *M. brassicae* damage nor by artificial wounding.

JA is a major signaling molecule involved in response to wounding and defense against chewing herbivores [58–60]. Concordantly, artificially wounded leaves showed upregulation of most of the genes involved in JA biosynthesis (i.e. *LOX2*, *AOS*, *AOC1* to *AOC4*, *OPR3*), JA homeostasis and turnover (i.e. *JAZ2*, *JAZ9*, *JA10*, *IAR3*, *ILL6*, *CYP94B3*) [61–64] and JA signaling (i.e. *VSP1*, *VSP2*) [58]. The JA-responsive defensin *PDF1.2a* [65] was upregulated as well in artificially wounded leaves. Furthermore, several JA-responsive genes involved in biosynthesis (i.e. *CYP81F4*, *IGMT5*) [55, 66] and metabolism of glucosinolates (i.e. *PYK10*, *NSP1*) [67, 68] were upregulated.

### Prior cold treatment affects the transcriptional response to tissue damage

To analyze the influence of a preceding cold stress on the transcriptional response to artificial wounding or feeding by a generalist or specialist herbivore, transcriptome analyses of leaf material from plants subjected to the treatments described in the Methods section and in Fig. 1 were performed.

**Fig. 4:**
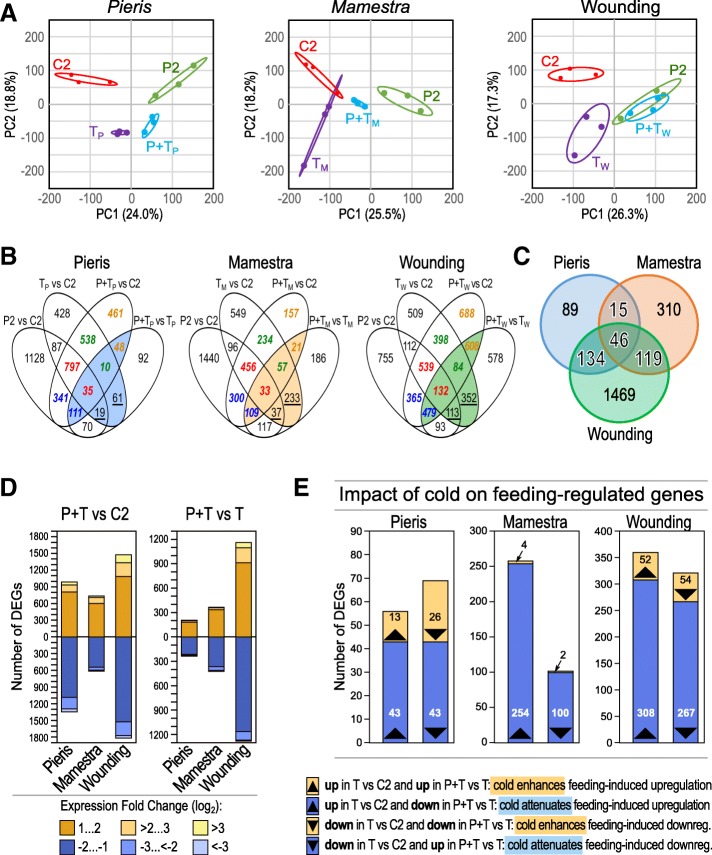
Previous cold treatment alters the transcriptional response to herbivory and artificial wounding. Leaves of *A. thaliana* were untreated (C2), cold-treated (P2), damaged by *Pieris brassicae* (T_P_) or *Mamestra brassicae* feeding (T_M_) or artificial wounding (T_W_), or cold-treated followed by feeding/wounding damage (P + T_P_, P + T_M_ and P + T_W_). *N* = 3 biological replicates of each sample type. **a** Principle component analysis (PCA) of the normalized gene expression of individual experimental leaf samples. The first two components, which explain most of the changes, are depicted (explained variances are shown at the axes). Ellipses indicate the 95% confidence interval. **b** Venn diagrams of genes regulated in response to larval feeding or artificial wounding with and without prior cold treatment. Blue characters, genes specifically regulated upon cold treatment; green characters, genes specifically regulated upon damage; red characters, genes regulated upon both cold per se and damage per se; orange characters, genes regulated only when the plant had been exposed to the combination of prior cold and subsequent damage; colored intersections, genes that were differentially regulated in P + T plants relative to T plants (P + T vs T) and also in untreated (T vs C2) or cold-treated, damaged plants (P + T vs C2) relative to control plants. **c** Venn diagram with the genes in the colored sectors in panel (**b**). **d** Numbers of differentially expressed genes (DEGs) in cold-treated and herbivore- or wounding-damaged plants compared to untreated plants (P + T vs C2; left panel) and in cold-treated herbivore- or wounding-damaged plants compared to untreated, damaged plants (P + T vs T; right panel). **e** Genes responsive to herbivory or artificial wounding with enhanced or attenuated expression changes in cold-treated relative to untreated plants

A principle component analysis of gene expression values revealed a clear separation of the C2 control plant transcriptome from that of the other plant treatments, except for the transcriptome of *M. brassicae* feeding-damaged plants (T_M_), whose 95% confidence interval overlapped slightly with that of the C2 control (Fig. 4a). The cold-treated plants (P2) showed a transcriptome shift relative to the C2 control, displayed in the first principle component (PC1), which accounts for ~ 25% of sample variances in all three sample groups. This indicates that deacclimation was not yet completed at the time of sampling. Subsequent feeding damage by *P. brassicae* (P + T_P_) or *M. brassicae* (P + T_M_) led to a separation of the transcriptome from that of P2 plants, whereas artificial wounding (P + T_W_) did not. This suggests that a prior cold treatment results in a different plant transcriptional response to continuous two-day-larval feeding damage than to discontinuous artificial wounding. Moreover, the T- and P + T-induced transcriptomes differed also in a species-specific manner, indicating that *Arabidopsis* can distinguish between damage by *P. brassicae* or *M. brassicae* (Additional file 1: Figure S3).

In cold-treated plants, the total number of regulated genes ranged from 1367 in *M. brassicae*-damaged leaves to 2341 in *P. brassicae*-damaged leaves to 3293 in artificially wounded leaves relative to untreated and undamaged control plants (Fig. 4b, d; P + T vs C2; the regulated genes are listed in Additional file 2: Table S1). Following prior cold stress, 446, 793, and 2439 genes were differentially regulated compared to untreated plants upon *P. brassicae* or *M. brassicae* feeding or artificial wounding, respectively (Fig. 4b, d; P + T vs T). Thus, the total number of genes which were differentially expressed due to prior cold stress was higher in artificially wounded than in larval feeding-damaged plants. In general, roughly equal fractions of DEGs were up- or downregulated in P + T_P_, P + T_M_, and P + T_W_ plants compared to the respective T plants.

Of particular interest are those genes that were differentially regulated in cold-treated and damaged plants relative to damaged plants (P + T vs T) and also in untreated and damaged (T vs C2) and/or cold-treated and damaged plants (P + T vs C2) relative to control plants (Fig. 4b, colored intersections in Venn diagrams). These gene sets comprise 284, 490, and 1768 genes in *P. brassicae* feeding-, *M. brassicae* feeding- and wounding-damaged leaves, respectively (Additional file 2: Table S1). The 80, 270, and 465 genes in the intersection of T vs C2 and P + T vs T, but not P + T vs C2 (Fig. 4b, underlined numbers) were regulated by tissue damage, however, the magnitude of the transcriptional response to damage was diminished when the plants had previously experienced cold. In contrast, genes exclusively occurring in the overlapping intersection of P + T vs T and P + T vs C2 were regulated only upon sequential experience of cold and tissue damage by feeding or wounding, but not by damage of untreated control plants. The intersections of T vs C2, P + T vs T and P + T vs C2 consist of genes that respond to feeding or wounding, and this response was significantly different when plants had been exposed to a prior cold phase.

We further investigated whether genes were specifically or commonly regulated by the three cold / damage combinations. Upon prior cold treatment, 46 DEGs were commonly regulated (40 up, 6 down), i.e. their transcriptional response was independent of the insect species and type of wounding (larval feeding, artificial damage) (Fig. 4c, Additional file 2: Table S1). Additionally, 15 genes were differentially regulated (7 up, 8 down) after cold exposure and subsequent feeding damage by both herbivore species, but not after cold exposure and subsequent artificial wounding (Fig. 4c).

The prior cold treatment also affected the magnitude of the transcriptional response to subsequent tissue damage. In *Pieris*-damaged leaves, the cold pre-treatment caused a significant intensification of damage-induced up- or downregulation in 39 of the 125 damage-induced genes (31%), whereas in the remaining genes the magnitude of regulation was diminished or even turned into opposite regulation (Fig. 4e; Additional file 2: Table S1). In artificially wounded local leaves, regulation of 84% of the damage-induced genes was attenuated. In leaves damaged by *M. brassicae,* almost all (98%) feeding-induced genes exhibited attenuated regulation upon prior cold treatment. Only 2% of the feeding-induced genes exhibited intensified expression changes in cold pre-treated plants (Fig. 4e). These results show that a cold phase attenuated the transcriptional response to subsequent leaf damage in the majority of damage-induced genes. However, the degree of attenuation was dependent on the type of damage.

### Leaf tissue damage affects the cold deacclimation process

**Fig. 5:**
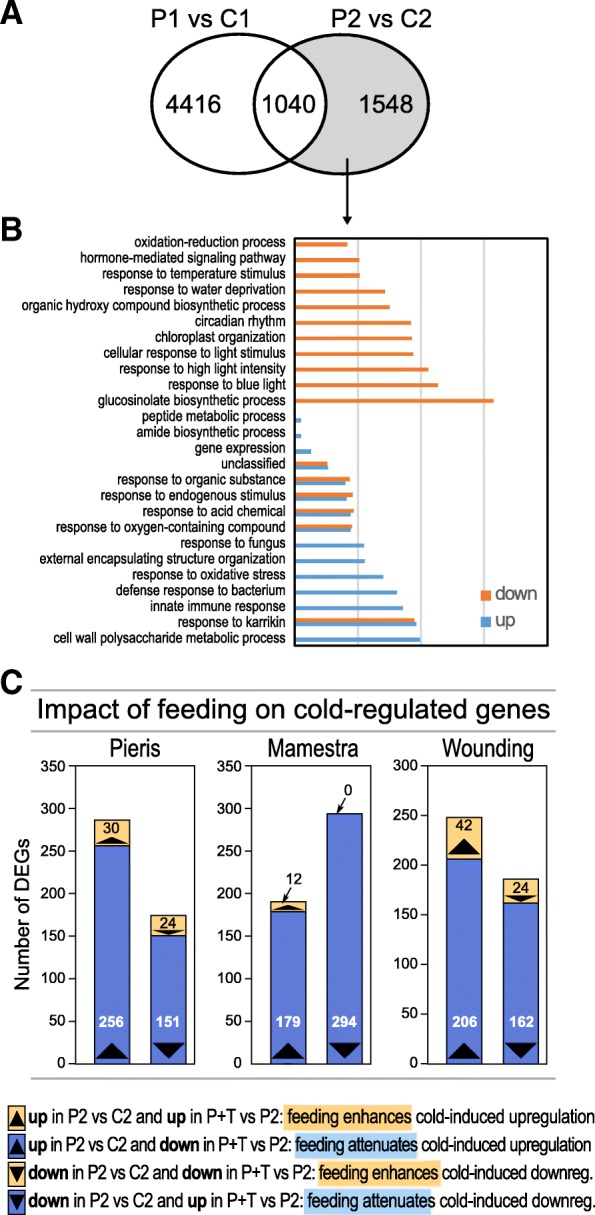
Cold deacclimation and impact of larval feeding or artificial wounding on cold-regulated genes. **a** Number of uniquely and commonly regulated genes after 5 days cold at 4 °C (P1) and after 3 days of cold deacclimation at 20 °C (P2) in comparison to the untreated controls (C1 and C2). **b** Enrichment of biological process gene ontology (GO) terms among the P2 vs C2-specifically up- and downregulated genes. **c** Cold-responsive genes with enhanced or attenuated expression changes in leaves exposed to larval feeding or artificial wounding leaves relative to leaves of undamaged plants

To investigate whether leaf tissue damage by larval feeding and artificial wounding has an impact on gene expression during deacclimation, we compared the transcriptomes of cold-treated plants during deacclimation with or without experience of tissue damage. First, we compared the transcriptome of plants at the end of the cold-period (Fig. 1; P1 plants) with that of plants after 3 days of deacclimation (Fig. 1; P2 plants). In the P2 plants we found more than 1500 newly regulated genes with 25 significantly enriched biological process GO terms, indicating that deacclimation also involves activation of cellular processes (Fig. 5a). Eleven GO terms are enriched only for downregulated genes, nine terms only for upregulated genes, and six terms are enriched for up- and downregulated genes (Fig. 5b). Interestingly, the downregulated terms include the ‘glucosinolate biosynthesis process’. A closer look reveals that in this category especially genes with function in aliphatic glucosinolate biosynthesis were downregulated, like *MAM3*, *CYP79F1*, *CYP79F2*, *SOT18*, *IPMI1*, *IPMI2 and CYP83A1.* Noticeably, with the exception of *MAM3*, none of these genes were differentially regulated after 5 days cold in P1 plants.

It was therefore interesting to investigate how larval feeding or wounding affects this cold deacclimation response, especially with respect to the genes involved in glucosinolate biosynthesis. Overall, the regulation of 14–19% of all 2588 DEGs in deacclimating P2 plants was attenuated when feeding or wounding occurred (Fig. 5c, Additional file 2: Table S1), resulting in a faster decay of the cold deacclimation response. However, feeding damage by *P. brassicae* larvae resulted in higher expression of five of the seven above mentioned aliphatic glucosinolate biosynthesis genes (*MAM3*, *CYP79F1*, *CYP79F2*, *SOT18* and *IPMI1*) in P + T_P_ compared to deacclimating P2 plants (expression of *IPMI2* and *CYP83A1* is not altered)*.* In contrast, feeding by *M. brassicae* larvae increased the expression of only two of the seven genes (*IPMI1* and *CYP79F1*). Wounding alone did not increase the transcription level of any of the seven genes.

### Stress- and stress combination-dependent transcriptional regulation of biological processes

**Fig. 6:**
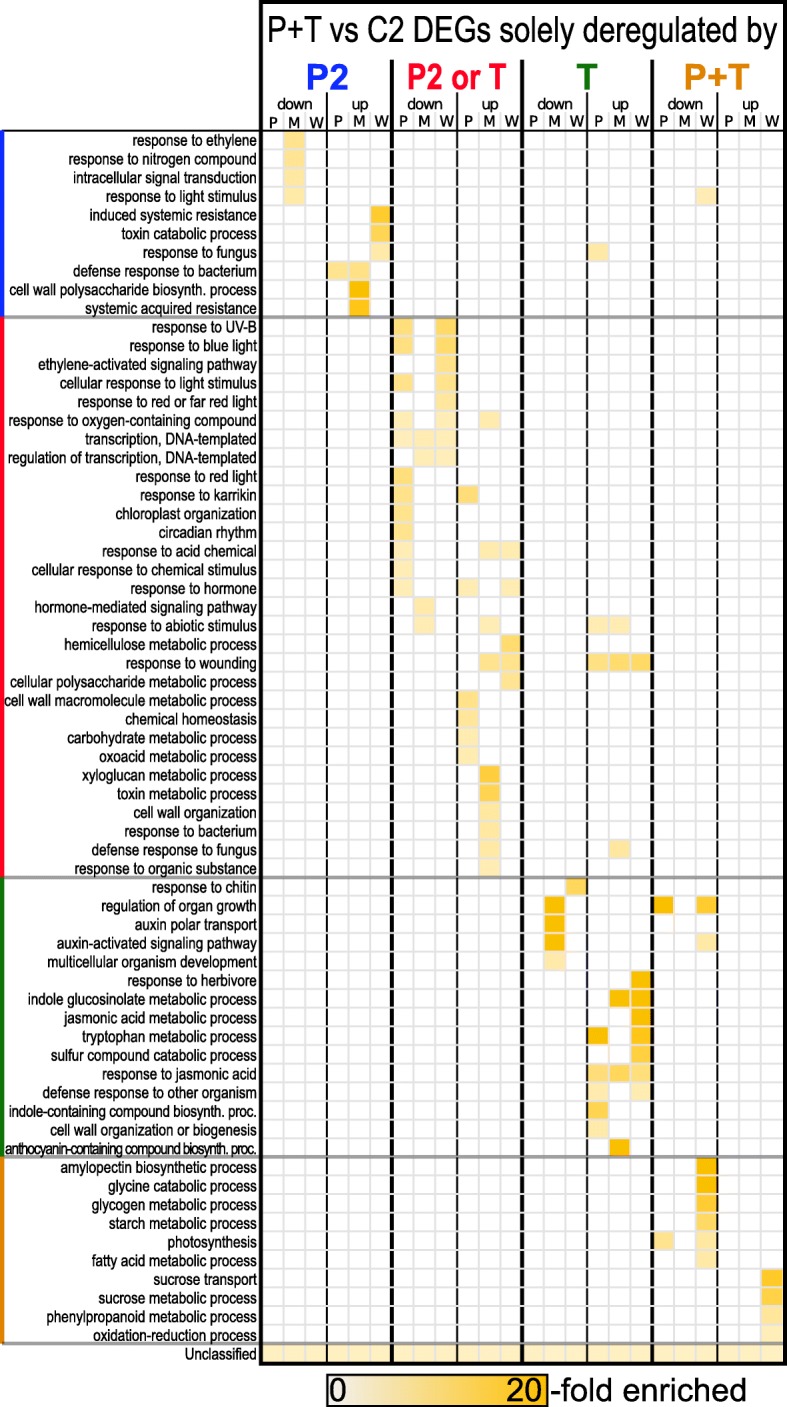
Treatment-specific enrichment of biological process GO terms of regulated genes in P + T plants. Fold enrichment of biological process GO terms in P + T samples relative to C2 (P + T vs C2) samples of DEG subgroups that are solely deregulated by cold (P2; corresponding to the sectors marked with blue characters in Fig. 4b), by either cold or feeding / artificial wounding (P2 or T; corresponding to the sectors marked with red characters in Fig. 4b), by larval feeding or artificial wounding (T; corresponding to the sectors marked with green characters in Fig. 4b), or only by the combination of cold and larval feeding or artificial damage (P + T; corresponding to the sectors marked with orange characters in Fig. 4b)

The transcriptome analyses revealed that (i) a preceding cold phase leads to a modified transcriptional response of feeding- or wounding-regulated genes (Fig. 4e) and (ii) leaf damage by feeding or wounding modifies the transcription profile of cold-regulated genes during deacclimation (Fig. 5c). This raised the question which biological process GO terms contributed to the overall transcriptional status of P + T plants. We thus determined the enriched GO terms (Fig. 6) among the genes differentially regulated solely by cold treatment (blue characters in Figs. 4b and 6), by damage (green characters in Figs. 4b and 6), by cold or damage (red characters in Figs. 4b and 6) and by the combination of prior cold and subsequent damage (orange characters in Figs. 4b and 6), respectively. Enhanced gene regulation in many biological process GO terms was triggered almost exclusively by the single stresses cold (P2), damage (T), or the combined stressors cold+damage (P + T). Other GO terms, though, were enriched in cold exposed plants but also after damage (P2 or T). For example, leaf damage exclusively contributed to upregulation of the ‘response to JA’ process. In contrast, in the process ‘response to wounding’ some genes were induced by cold or damage, while other genes were upregulated only by damage.

Of all regulated genes in P + T plants, 13% (*Mamestra*), 22% (*Pieris*) and 39% (Wounding) only changed in expression if a cold treatment preceded the tissue damage (Fig. 4b, orange characters). These genes can be considered as primable for improved damage-triggered induction by prior cold exposure.

Damage of *A. thaliana* leaves by *P. brassicae*, *M. brassicae* and artificial wounding resulted in significant transcriptional changes in 187, 173, and 235 defense- or glucosinolate synthesis-related genes (Additional file 2: Table S1). Of these, 30% (*Pieris*), 43% (*Mamestra*) and 30% (Wounding) were upregulated. Remarkably, 70% of the defense- or glucosinolate synthesis-related genes upregulated by *P. brassicae* feeding were also upregulated by *M. brassicae* feeding, whereas 75% of the genes downregulated upon *P. brassicae* feeding were not regulated by *M. brassicae* feeding. When preceded by cold, the transcriptional response of only 4% of these *P. brassicae* feeding-induced genes was attenuated by ≥2-fold. In contrast, responses of much greater fractions of genes deregulated by *M. brassicae* feeding (29%) or wounding (32%) were attenuated by a factor of 2 or more, when the plants had previously been exposed to cold.

Thus, drought or cold stress preceding tissue damage apparently affects similar biological processes of *A. thaliana*, but not necessarily the same genes.

## Discussion

### Similarities and differences in *A. thaliana* transcriptional response to leaf damage by *P. brassicae* feeding, *M. brassicae* feeding and artificial wounding

After a prior cold treatment of *Arabidopsis* plants, larvae of the generalist herbivore *M. brassicae* performed better than on untreated plants whereas larvae of the specialist *P. brassicae* did not benefit, indicating that the cold treatment induced changes in the plant that promoted larval development of *M. brassicae*, but not of the specialist *P. brassicae*. It is conceivable that after a cold phase the plant’s metabolic status or response to differences in leaf tissue damage patterns is altered.

For each of the three leaf damage scenarios approximately two thirds of all regulated genes were also regulated in one or both of the other two damage types. Remarkably, > 98% of these genes were regulated in the same direction, only 29 genes showed opposite regulation upon different damage types (Fig. 3b). Several of the genes with the largest regulation differences are known to be involved in plant responses to phytopathogens. For example, *argah2* mutants show increased susceptibility to pathogens inducing clubroot disease [69, 70]. SLAH3 is an anion channel expressed in guard cells and involved in stomatal immunity by closure of guard cells in response to pathogen attack [71]. DREB26 is responsive to infection by the necrotrophic fungus *Botrytis cinerea* and to various abiotic stresses as well [72]. PXMT1 is a target of miR163, a microRNA which promotes in a light-dependent manner seed germination and primary root length [73] and modulates defense responses against bacterial pathogens [74]. The Aux/IAA protein IAA29 is a transcription factor acting as repressor of the auxin signaling pathway [75].

Several studies addressed the hypothesis that highly specialized herbivores are more tolerant towards defenses of their host plants than generalists (reviewed by [46]). However, plant defense responses are multifaceted and not by default more effective against generalists than specialists. Thus, to identify plant responses specifically induced or suppressed by generalist and specialist herbivores, a treatment like artificial wounding can provide a baseline for changes at the molecular level [46, 76]. Here, 23–30% of genes transcriptionally responding to leaf damage by the specialist *P. brassicae*, the generalist *M. brassicae* or artificial wounding were shared (Fig. 3b, central intersection), including upregulation of JA-responsive defense-related genes. This shows that part of the responses to feeding damage overlapped with the reaction to artificial wounding. The majority of wound-responding genes could not be assigned to a distinct, significantly regulated process, indicating a generic “panic” response of *A. thaliana* to artificial wounding [77].

Artificial wounding resulted in upregulation of many JA biosynthesis genes. Most of these genes were also upregulated to very similar levels in response to feeding by *M. brassicae* larvae, whereas transcriptional induction was attenuated or lacking upon feeding by *P. brassicae* larvae (Additional file 2: Table S1). Interestingly though, many SA-responsive genes are downregulated in response to *P. brassicae* feeding. Salicylic acid can act antagonistically to JA-mediated plant defense responses [12, 14, 60, 78], but it can also positively modulate the plant defense against herbivores [35, 79]. We found that after two days feeding by 10 *P. brassicae* larvae eight SA-associated WRKY transcription factors are downregulated. The SA-responsive factors *WRKY38*, *WRKY60* and *WRKY70* are only downregulated upon *P. brassicae* feeding, but not upon *M. brassicae* feeding or artificial wounding. It will thus be interesting to investigate whether *P. brassicae* oral secretions negatively affect the plant’s SA-response pathway towards a diminished herbivore defense [80–82].

Strikingly, opposite to *P. brassicae* feeding, *M. brassicae* feeding was accompanied by more up- than downregulated SA-response genes, and seven of the eight *WRKY* genes downregulated upon *P. brassicae* feeding were not responding to *M. brassicae* feeding. It is tempting to speculate that, in contrast to *P. brassicae*, *M. brassicae* oral secretions do not dampen the plant’s SA-response pathway. Moreover, *M. brassicae* feeding induced in *Arabidopsis* leaves a stronger and more complex transcriptional response of glucosinolate biosynthesis-associated genes. Striking is the upregulation of *MYB51*, a regulator of indole glucosinolate biosynthesis [83], the nitrile specifier protein *NSP1* [84], the P450 monooxygenases *CYP81F2* [85] and *CYP81F4* [66] and the indole glucosinolate methyltransferase *IGMT2* [55]. Elevated expression of plant specifier proteins has been found to promote *A. thaliana*’s defense against *P. rapae* larvae*,* a close relative of *P. brassicae,* as it deters *P. rapae* from egg deposition on the plants. In addition, the endoparasitoid *Cotesia rubecula*, which prefers *P. rapae* larvae as hosts, is more attracted to *P. rapae*-infested plants overexpressing specifier proteins than to *P. rapae*-infested Col-0 wild-type plants. In contrast to Col-0, the specifier overexpressors accumulate mainly simple nitriles from glucosinolate hydrolysis [86]. *CYP81F2* encodes a P450 monooxygenase involved in 4MI3G (4-methoxyindol-3-ylmethylglucosinolate) synthesis and antifungal defense [85].

Studies of plant interactions with the lepidopteran generalist *Spodoptera littoralis* and with specialists (including *P. rapae* and *P. brassicae*) revealed that application of larval oral secretion of these insects results in suppression of plant defense gene expression [87, 88]. Among the genes with attenuated expression in our study the protease inhibitor DR4 and extracellular lipase 3 EXL3 showed a more pronounced attenuation upon *P. brassicae* than upon *M. brassicae* feeding (Additional file 2: Table S1). These genes are also suppressed upon feeding by *S. littoralis* [87]. It is thus conceivable that the expression attenuation we observed was caused by oral secretions of the herbivores. It is known that plants can distinguish between damage by different herbivores and by artificial wounding [89] because their oral secretions contain species-specific herbivore-associated molecular patterns (HAMPs) that enable plants to modulate their defense responses (reviewed in [90–92]). It will be interesting to investigate in the future whether the observed differences between the expression patterns upon *P. brassicae* or *M. brassicae* feeding depend on such HAMPs.

### Prior low temperature exposure causes attenuated regulation of genes responsive to leaf damage

The comparison of expression changes in herbivory- or wounding-responsive genes in plants, which had previously experienced 5 days at 4 °C, revealed similarities, but also striking damage type-dependent differences in transcriptional reprogramming. Among the 46 genes that were regulated after each of the three damage types, several were reported to function in stress responses. The flavonol monooxygenase 1 (*FMO1)* is known to be essential for the establishment of systemic acquired resistance (SAR) and therefore systemic defenses against pathogens like *Pseudomonas syringae* [93]. *UGT72E2* and *UGT72E3* are involved in glucosylation of monolignols, which results in increased content of coniferin, syringin, and other phenylpropanoids [94–97]. ALLENE OXIDE CYCLASE1 (*AOC1),* a key enzyme in JA biosynthesis, is known to be rapidly responding to cold stress ([98, 99], reviewed by [100]).

Fifteen genes were differentially regulated only upon leaf damage by either of the two herbivores but not upon artificial wounding. Among the eight commonly downregulated genes is a terpene synthase (*TPS03*), which is known to be inducible by wounding and herbivory [101]. The transcription factors *RAP2.9* and *ZAT10* function as regulators in biotic and abiotic stress responses as well as in stress combinations [102–104]. *ORA59* is involved in JA/ET synergistic regulation and important for pathogen defense via *PDF1.2* activation [15, 105]. Commonly upregulated genes (7) include *LOX5,* a member of the 9-lipoxygenases involved in pathogen defense [106] and PIL1, a transcription factor known to be cold- and high light-stress responsive with functions in shade avoidance. It is also JA responsive in a COI-dependent manner [107–109]. The gene *ST2A* displayed increased expression in previously cold-experienced plants responding to *P. brassicae* feeding, while the response to *M. brassicae* was opposite. ST2A, one of 18 sulfotransferases in *Arabidopsis*, is involved in JA metabolism by sulfating 11-OH-JA and 12-OH-JA [110].

Common for all three types of tissue damage was that a smaller fraction of damage-responsive genes was more strongly up- or downregulated, whereas in the majority of them the transcriptional response was attenuated after a prior cold treatment. The difference in the fractions of genes with altered regulation between *P. brassicae* and *M. brassicae* is striking, though. In leaves damaged by *P. brassicae* larvae 31% of the genes are more intensely and 69% more weakly regulated. In contrast, upon herbivory by *M. brassicae* larvae, only 2% of the damage-responsive genes are more intensely regulated whereas in 98% of these genes the expression change is lower than in plants that were not exposed to cold.

### Feeding and wounding promote a decline of the cold acclimation status

Cold acclimation and subsequent deacclimation are known to be accompanied by extensive transcriptomic and metabolomic reorganization. Not only acclimation but also deacclimation is an active and tightly regulated process, which involves metabolic changes in lipid and cell wall components, downregulation of protein synthesis, and transcriptional reprogramming of jasmonate, brassinosteroid and other hormonal pathways [111, 112]. Pagter et al. [111] found that the deacclimation-associated responses of *A. thaliana* Col-0 proceed most rapidly during the first 12 h after shifting 4 °C-acclimated plants to 20 °C. However, deacclimation is only in part a reversion of cold acclimation, and even after 24 h the plant metabolism and transcriptome have not yet fully reverted to the non-acclimated status [111]. It is thus conceivable that after 24 h of deacclimation the plant response to herbivore attack differs from that of untreated plants, but it is not predictable whether the prior cold treatment results in an unspecific or herbivore-specific, improved or compromised defense.

Although the cold deacclimation response is considered to be rapid and mainly passive [112, 113], more than 1500 genes were newly regulated 3 days after terminating the plant’s exposure to cold. Similar results were obtained in an earlier study by Firtzlaff et al. [43]. Conspicuously, among the newly regulated genes the GO term ‘glucosinolate biosynthesis process’ is downregulated. A weaker expression of these genes could imply a reduced aliphatic glucosinolate content in P2 plants and therefore provide advantageous conditions for the larvae of the generalist herbivore species, *M. brassicae.* Performance of this generalist species is negatively affected by aliphatic glucosinolates [114, 115]. In contrast, the specialist *P. brassicae* is well known to effectively detoxify glucosinolates (e.g. [48]).

In addition, the differences in the transcriptional response of cold-treated *Arabidopsis* plants to feeding by the two herbivores support the notion that the generalist *M. brassicae*, but not the specialist *P. brassicae*, benefits from a cold phase prior to hatching of the larvae. For instance, *AOS* (allene oxide synthase), a key gene in JA biosynthesis [116], the antifungal/antimicrobial defense thionin gene *THI2.1* [117], and the indolic glucosinolate synthesis genes *CYP81F4*, *CYP81F2* and *IGMT1* [55, 66, 118] were induced in plants not exposed to cold by *M. brassicae* feeding, but not by *P. brassicae* feeding. In cold-treated plants, expression of these genes was attenuated upon *M. brassicae* feeding, but not altered upon *P. brassicae* feeding. This is consistent with the observation that the performance of *P. brassicae* larvae is identical on cold-treated and control plants, whereas *M. brassicae* larvae perform better on cold-treated plants. Yet, the plant’s defense response invoked by the feeding damage of *P. brassicae* larvae is comparable in untreated and cold-treated *A. thaliana* plants. Since the specialist *P. brassicae* is well adapted to the defense measures [119, 120] it was expected that its performance is not impaired.

Since *M. brassicae* is more sensitive to the defense compounds of *A. thaliana* [114], its performance in untreated plants is negatively affected. In cold-treated plants, though, the *M. brassicae* feeding damage pattern elicited an attenuated defense reaction. These results are in accordance with two other studies that addressed the question of how the experience of prior abiotic stress influences later defense responses against herbivores [42, 43]. Common results of the three studies are: (i) prior exposure of plants to abiotic stress caused a reduced transcriptional induction of tissue damage-inducible defense genes, including attenuated gene expression of e.g. JA- and glucosinolate metabolism-related genes; (ii) the performance of the specialist herbivores *P. rapae* [42] and *P. brassicae* (this study and [43]) was not affected by prior drought or cold treatment of *A. thaliana*; (iii) herbivory led to a shift from the drought- or cold-adapted transcriptome towards herbivore defense, thus accelerating the abiotic stress deacclimation. Yet, the differentially regulated genes in feeding-damaged plants with prior drought or cold experience differed to a great extent. Only two genes, a glutathione S-transferase (*GSTU8*) and *UPF0496* were transcriptionally responding to all tissue damage types when preceded by drought or cold.

## Conclusions

We show that a prior cold treatment of *A. thaliana* differentially reprogrammed the transcriptional response to leaf tissue damage by artificial wounding and feeding by the specialist herbivore *P. brassicae* or the generalist herbivore *M. brassicae*. The cold-treatment resulted at the transcriptional level in an attenuation of the plant’s damage-induced defense response. We suggest that this attenuation is responsible for the improved larval performance of the generalist *M. brassicae*. In contrast, the specialist *P. brassicae* is unaffected by the damage-induced *A. thaliana* defense measures and accordingly does not benefit from the defense attenuation by a preceding cold treatment of the plants.

## Methods

### Plant growth

*Arabidopsis thaliana* Columbia Col-0 seeds (Stock No. N1093) were obtained from the Nottingham Arabidopsis Stock Centre (NASC). Seeds were sown on soil type A (2:2:1, Einheitserde CL P: Einheitserde CL T: Sand) and stratified for 2 days at 4 °C. Thereafter, plants were grown in a growth chamber at short day conditions (8 h/16 h light/dark cycle, 120μE), 20 °C and 50% relative humidity for 7 weeks. Three-week-old seedlings were transplanted in pots containing soil type B (7:7:3, Einheitserde CL P: Einheitserde CL T: Perlite).

### Insect rearing

*Pieris brassicae* larvae from in-house captive breeding were reared on savoy cabbage (*Brassica oleracea* convar. *Capitata* var. *sabauda*) as described by [43]. *Mamestra brassicae* were obtained from N. Fatouros (Biosystematics Group, Wageningen University and Research, Wageningen, Netherlands). Larvae were reared on cabbage plants (*Brassica oleracea* var. *sabellica* L.) until pupation. Soil was provided to last instar *M. brassicae* larvae for pupation, while *P. brassicae* pupae were kept on cardboard. Adults of *M. brassicae* were offered water and a sugar-water solution (1:5 w/v). Adult *P. brassicae* butterflies were fed with an aqueous honey solution.

### Plant treatments

The experimental design is depicted in Fig. 1. Seven-week-old plants were subjected to (i) 5 days cold at 4 °C (P samples), (ii) leaf damage by *P. brassicae* larvae (T_P_ samples), *M. brassicae* larvae (T_M_ samples) or artificial wounding (T_W_ samples), (iii) cold followed by leaf damage (P + T_P_, P + T_M_ or P + T_W_ samples), or (iv) no stimulus (C samples). The stimulus ‘cold’ was applied for 5 days, followed by 1 day under normal growth conditions (20 °C) as memory/deacclimation phase. P1 samples were taken directly after 5 days of cold and P2 samples 3 days after transferring plants back to 20 °C (Fig. 1). The second stress (larval herbivory or artificial wounding) was applied for 2 days following the 1 day memory/deacclimation phase. For treatment with larvae, neonate *P. brassicae* or *M. brassicae* larvae were added in a clipcage to leaf number 17. For control, an empty clipcage was placed on leaf number 17 of untreated control (C) and cold-pretreated (P) plants. Artificial wounding was applied by damaging leaf number 17 with forceps for 30 s two times a day for 2 days. The damaged area almost matched the area of damage that larvae feeding inside a clipcage inflicted to a leaf.

### Larval performance measurement

Individual seven-week-old plants treated with or without prior cold were subjected to feeding by 15 freshly hatched *M. brassicae* or *P. brassicae* larvae on leaf 17. The experiments were repeated 11 times (*N* = 11 plants) with *M. brassicae* and 15 times (*N* = 15 plants) with *P. brassicae*. Larvae were confined in clipcages with a diameter of 3 cm. Two days later, larval weight and weight gain were determined. Furthermore, the consumed leaf area was assessed by comparing pictures of the leaves taken before and after 2 days feeding using ImageJ [121]. The leaf expansion during the 2 days feeding period was marginal and not taken into account. Subsequently larvae were returned to the plants and allowed to feed upon the whole plant for another 4 days. Two and 4 days later larval weight and weight gain were measured again. Larval performance data were evaluated with “R” [122] and subjected to statistical analysis [123, 124]. Data were tested for normal distribution (Shapiro-Wilk test) and homogenous variances (Levene’s test). If larval weight and weight gain values were not normally distributed and/or did not show variance homogeneity, data were log_2_ transformed to fulfil the prerequisites for applying unpaired Student’s *t*-test.

### Transcriptome analyses

We analyzed the transcriptome of untreated plants (C), cold-exposed plants (P), damaged plants (T) and cold-exposed and feeding-damaged plants (P + T). We standardized the extent of damage by insects and artificial wounding to be able to ascribe damage-induced transcriptomic changes to the type of damage rather than to the extent of damage. Therefore, plant leaves were exposed to 10 *P. brassicae* larvae or 20 *M. brassicae* larvae in a clip cage. After 2 days feeding, the leaf area consumed by the two species was almost identical (Additional file 1: Figure S4). The artificially wounded area was similar as well. For RNA extraction, a 1 cm wide strip from leaf number 17 of C1, C2, P1, P2, T_P_, T_M_, T_W_, P + T_P_, P + T_M_ and P + T_W_ plants was harvested. The stripe was located proximal to the clipcage or wounding site. To minimize effects of circadian clock-dependent transcriptional regulation, all samples were collected 4 to 6 h after the onset of the daylight phase, i.e. at a time when larvae are actively feeding in nature. After harvesting, the strips were kept frozen in liquid nitrogen. Leaf material of three individual plants was pooled to obtain one biological replicate, and three biological replicates of each sample type were analyzed.

Frozen leaf material was ground in liquid nitrogen, and total RNA was extracted according to Onate-Sanchez [125]. Total RNA was DNase I-digested according to manufacturer’s instructions (Thermo Fisher Scientific). Yield and quality of extracted RNA was determined spectrophotometrically and by denaturing agarose gel electrophoresis.

Genome-wide expression analyses were conducted on ArrayXS Arabidopsis v2 microarrays (series XS-5010; GEO accession GPL19779; Oaklabs GmbH, Hennigsdorf, Germany). Microarray data were processed and analyzed with the *Bioconductor Linear Models* for microarray data (limma) software package [126, 127] as described in Firtzlaff et al. [43]. In short, microarray signals were background-corrected and interarray-normalized. Genes with ≥2-fold expression change and adjusted *P*-values ≤0.05 (Benjamini and Hochberg false discovery rate procedure) were defined to be differentially expressed genes (DEGs) (Additional file 1: Figure S2). Gene expression data are deposited in the NCBI GEO repository under the accession number GSE114211.

Principle component analysis (PCA) of the transcriptomic data sets was performed using the “ggplot” and “ggbiplot” packages of “R” [122, 128]. Enriched gene ontology (GO) terms were identified using the *TAIR GO Term Enrichment for Plants* tool (www.arabidopsis.org) provided by PANTHER DB (http://pantherdb.org). If not mentioned otherwise, a Bonferroni correction for multiple testing was applied to reduce false positives.

## Additional files

Additional file 1: **Figure S1.** Relative growth rates of *Pieris brassicae* and *Mamestra brassicae* neonate larvae on previously cold-treated or untreated plants. **Figure S2.** Gene expression changes in plants exposed to larval feeding or artificial wounding compared to untreated control plants. **Figure S3.** Principle component analysis of transcriptomes of plants exposed to individual treatments. **Figure S4.** Leaf area consumption by *Pieris brassicae* and *Mamestra brassicae* neonate larvae after 2 days feeding upon previously cold-treated or untreated plants. (PDF 348 kb)

Additional file 2: **Table S1.** List of genes differentially expressed in response to cold treatment and/or feeding by *Pieris brassicae* larvae, feeding by *Mamestra brassicae* larvae or artificial wounding. (XLSX 1843 kb)

## Data Availability

Microarray transcription raw data are deposited in the NCBI Gene Expression Omnibus (GEO) repository under the accession number GSE114211.

## Abbreviations

ABA: Abscisic acid
DEG: Differentially expressed gene
ET: Ethylene
GO: Gene ontology
JA: Jasmonic acid
PCA: Principal component analysis
SA: Salicylic acid
