# Supplementary material for: The differential response of cold-experienced Arabidopsis thaliana to larval herbivory benefits an insect generalist, but not a specialist
Source: BMC Plant Biol. 2019 Aug 2;19:338. doi: 10.1186/s12870-019-1943-3 (PMC6679549; doi:10.1186/s12870-019-1943-3)
Supplement: Supplementary file 1 — Figure S1. Relative growth rates of Pieris brassicae and Mamestra brassicae neonate larvae on previously cold-treated or untreated plants. Figure S2. Gene expression changes in plants exposed to larval feeding or artificial wounding compared to untreated control plants. Figure S3. Principle component analysis of transcriptomes of plants exposed to individual treatments. Figure S4. Leaf area consumption by Pieris brassicae and Mamestra brassicae neonate larvae after 2 days feeding upon previously cold-treated or untreated plants. (PDF 348 kb) [file 12870_2019_1943_MOESM1_ESM.pdf]

Figure S1

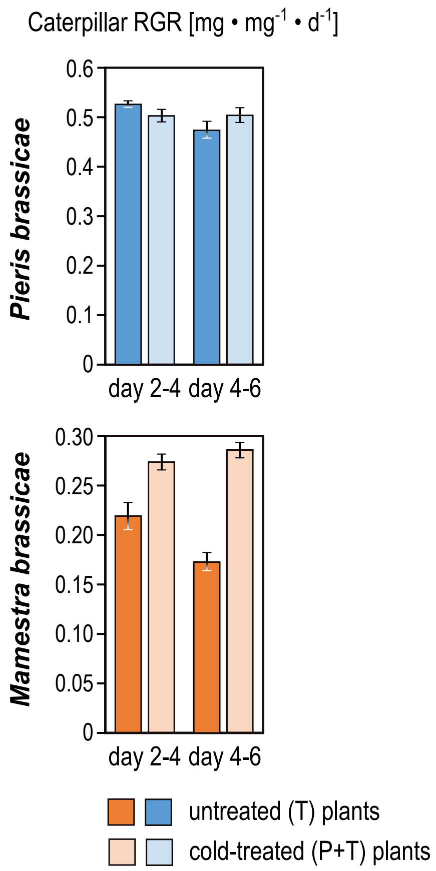

Figure S1: Relative growth rates of *Pieris brassicae* and *Mamestra brassicae* neonate larvae on previously cold-treated or untreated plants. Relative growth rates of larvae between day 2-4 and day 4-6 were calculated as [ln(weight day 4)-ln(weight day 2)/2 days] and [ln(weight day 4)-ln (weight day 2)/2 days] (mean values ±SE). N (*P. brassicae*): T=14 plants, P+T=14 plants; N (*M. brassicae*): T=11 plants, P+T=11 plants.

Figure S2

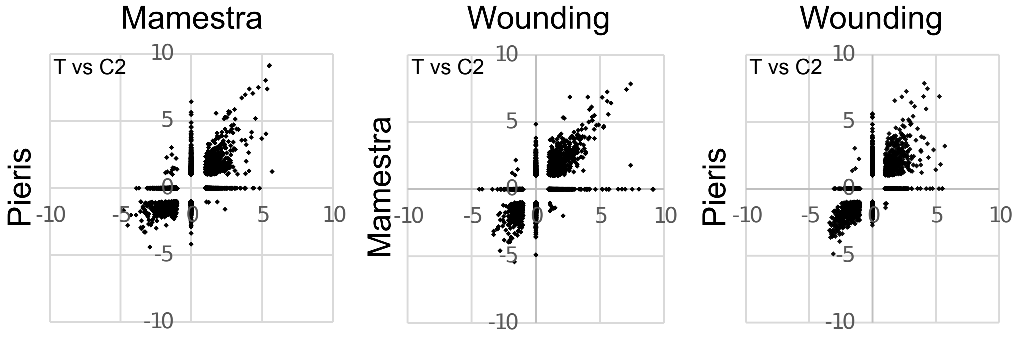

Figure S2: Gene expression changes in plants exposed to larval feeding or artificial wounding when compared to untreated control plants. The figure relates gene expression changes (T vs C2) in plants exposed to *Pieris brassicae* or *Mamestra brassicae* (left panel), *M. brassicae* or artificial wounding (center panel) and *P. brassicae* or artificial wounding (right panel).

Figure S3

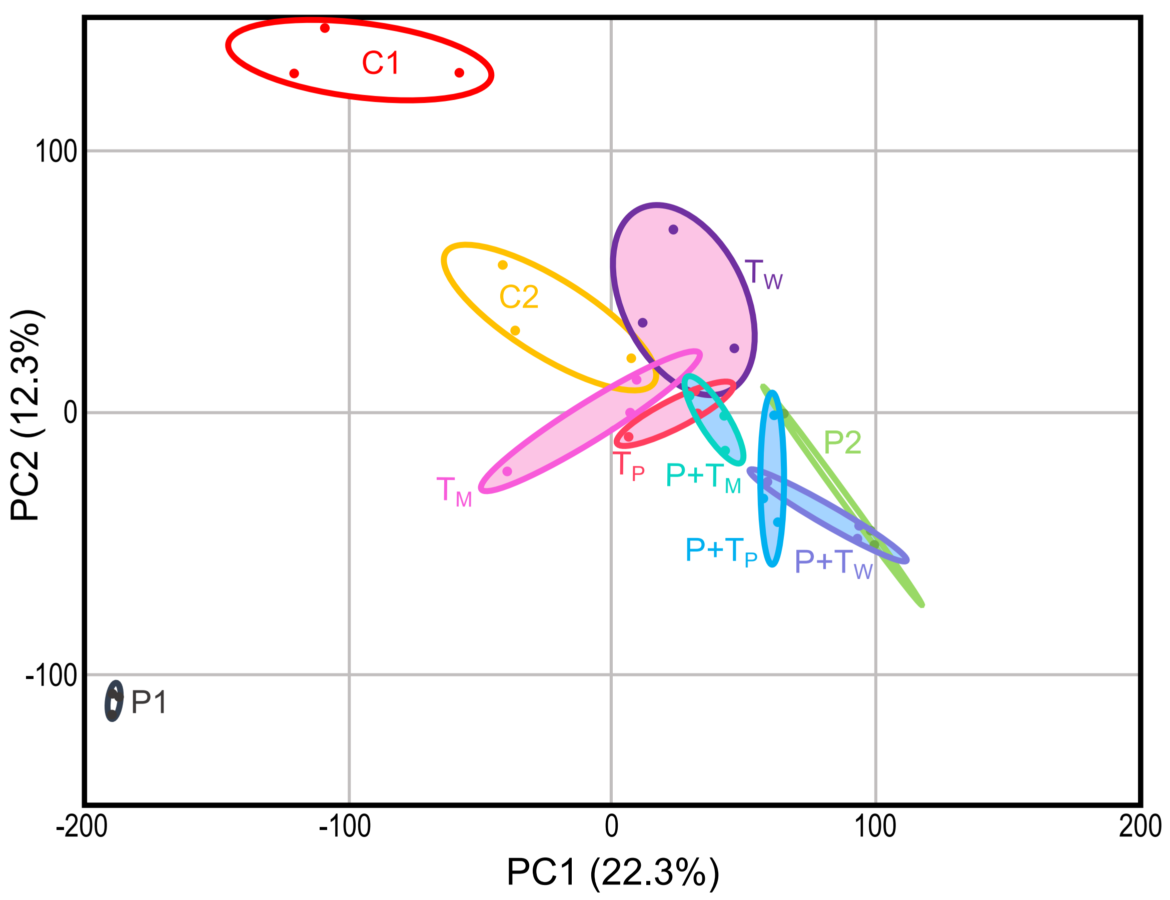

Figure S3: Principle component analysis of transcriptomes of plants exposed to individual treatments. Samples are designated according to Figure 1. N = 3 biological replicates of each sample type. The first two principal components, which explain most of the changes, are depicted. Ellipses indicate the 95% confidence interval.

Figure S4

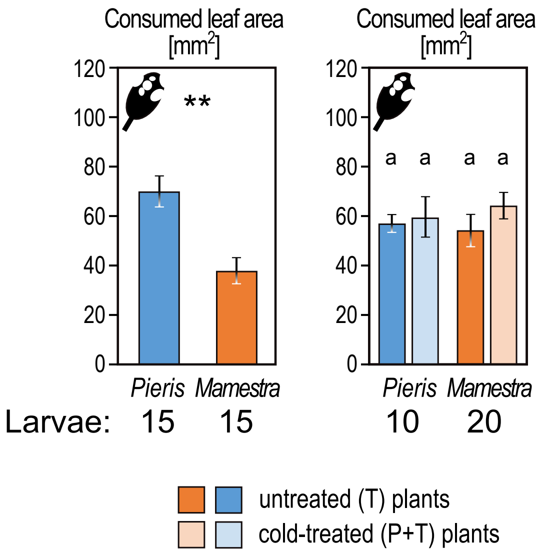

Figure S4: Leaf area consumption by Pieris brassicae and *Mamestra brassicae* neonate larvae after 2 days feeding upon previously cold treated or untreated plants. Larvae were placed onto plants as neonates. The number of larvae, which were placed in a clipcage attached to leaf 17 of a plant, is given on the X-axis. Bars show the consumed leaf area per plant after 2 days feeding (N = 3-5 plants; mean ± SE). Asterisks indicate significantly different ($P < 0.01$, Student's t-test) leaf areas consumed by 15 *Pieris brassicae* and 15 *Mamestra brassicae* larvae. Leaf areas consumed by 10 *P. brassicae* and 20 *M. brassicae* larvae do not significantly differ (one way ANOVA).
